# Effects of Iron Minerals on the Compressive Strengths and Microstructural Properties of Metakaolin-Based Geopolymer Materials

**DOI:** 10.3390/gels8080525

**Published:** 2022-08-22

**Authors:** Dimace Lionel Vofo Ngnintedem, Marco Lampe, Hervé Kouamo Tchakouté, Claus Henning Rüscher

**Affiliations:** 1 Laboratory of Analytical Chemistry, Department of Inorganic Chemistry, Faculty of Science, University of Yaounde I, Yaounde P.O. Box 812, Cameroon; 2 Institut für Mineralogie, Leibniz Universität Hannover, Callinstrasse 3, D-30167 Hannover, Germany

**Keywords:** hematite, magnetite, goethite, metakaolin, geopolymer materials, gels

## Abstract

The current study aims to investigate the influence of iron minerals on the amorphous phase content, compressive strengths and the microstructural properties of the geopolymer materials. Geopolymer materials were prepared by the substitution of metakaolin by 10 and 20 wt.% of each iron mineral sample. Sodium waterglass from rice husk ash was used as a hardener, and metakaolin was used as an aluminosilicate source. The X-ray patterns show that the iron minerals denoted FR and FB are associated with hematite and magnetite, respectively. FY contains goethite together with a significant content of kaolinite and quartz. It is observed in the XRD patterns and FTIR absorption spectra that the additions of hematite, magnetite and goethite remain largely unreacted in the geopolymer binder. The compressive strengths of the related geopolymer composites show some significant variations indicating certain effects for mechanical stability obtained: 10 wt.% replacement of metakaolin by hematite increased the compressive strength from 51.1 to 55.5 MPa, while 20 wt.% hematite caused a decrease to 44.9 MPa. Furthermore, 10 and 20 wt.% replacement with FB revealed decreased values 47.0 and 40.3 MPa, respectively. It was also found that 10 and 20 wt.% of FY caused lower values of 30.9 and 39.1 MPa, respectively. The micrographs of geopolymer materials present some voids and cracks. The denser matrix is related to a superior gel formation producing a better glue between the crystalline additions. The unsubstituted geopolymer sample provides with about 50% the highest X-ray-amorphous content, whereas the substituted samples range between 35 and 45%, indicating systematically smaller gel contents without any clear trend with the compressive strength variation, however. The strength dependencies reveal more complex interaction between the gel and crystalline additions.

## 1. Introduction

Iron is found mainly in the Earth’s crust in minerals of iron oxide and iron oxyhydroxide such as hematite, magnetite, maghemite, goethite, limonite, etc. Amongst these minerals, hematite (α-Fe_2_O_3_) and magnetite (Fe_3_O_4_) are mostly used as an ore for making metal iron. Iron minerals exist to some extent in aluminosilicate sources such as kaolin and more significantly in laterite. Most iron minerals generally present in these aluminosilicate sources are hematite and goethite. The iron mineral content in the aluminosilicate sources depends on the geological formation. Goethite contained in the raw aluminosilicates are transformed to hematite during the calcination process, which is performed at about 700 °C using these sources for the preparation of geopolymers. Several researchers have used hematite and magnetite nanoparticles as additives to increase the hydration process of the Portland cement mortar and concrete. For example, Kani et al. [1] investigated the effects of nano-hematite on the mechanical performance of the hardened Portland cements. They reported that the compressive and flexural strengths are higher when 3 wt.% of nano-Fe_2_O_3_ was added. The improved mechanical properties were related to the fact that the addition of nano-Fe_2_O_3_ reduces the size and the quantity of Portlandite crystals in the system, therefore filling the voids of the networks of calcium silicate hydrate. Yazdi et al. [2] studied the effect of Fe_2_O_3_ nanoparticles on the morphological properties and microstructure of cement mortar. They reported that the presence of 5 wt.% of Fe_2_O_3_ nanoparticles in the structure of Portland cement made mortars completely filled the pores, limited the formation of larger crystal sizes of Ca(OH)_2_, and made the matrix of the hydration products more homogeneous and denser. Ghazanlou et al. [3] showed in a comparative study on the mechanical, physical and morphological properties of cement-micro/nano Fe_3_O_4_ composites, that cement pastes reinforced by magnetite nanoparticles of sizes 20–40 nm had the highest compressive, flexural and tensile strengths, while the effectiveness of other Fe_3_O_4_ fillers of 80–100 nm, 250–300 nm and 1–2 µm were negligible. These authors argued that magnetite nanoparticles alter the loose needle-like microstructure of the hardened cement into a compact integrated morphology, which hindered crack propagation by toughening mechanisms (crack pinning, crack deflection and crack branching).

In this sense the addition of magnetite or hematite could result in a stabilisation of the gel for improved mechanical properties formed in Portland cement-based concretes and mortars. Thus, an interesting question is how this type of minerals affects the mechanical properties of geopolymer materials suggested as an environmentally friendly substitute for Portland cement. The setting and hardening of geopolymer materials are due to the formation of a macromolecule by the polycondensation reaction between the silicate and the aluminate species. Essaidi et al. [4] studied the role of hematite on the properties of metakaolin-based geopolymer materials. They concluded that the addition of hematite in the geopolymer systems decreases the mechanical properties owing to their friable character, which is displayed in the structure of geopolymer materials. On the other hand, Zailani et al. [5] investigated the effect of hematite on the properties of fly-ash-based geopolymer. They concluded that the higher content of Fe_2_O_3_ in the raw material could improve the overall properties of geopolymer materials. Kaya et al. [6] studied the influence of the micro Fe_2_O_3_ on the physical and mechanical properties of zeolite-and-kaolin-based geopolymer mortar. They reported that the incorporation of micro Fe_2_O_3_ in the zeolite-based geopolymer improves their physical and mechanical properties. Abdalla et al. [7] studied the influence of nano-hematite on the properties of Portland cement and geopolymer concrete. The findings of these works reveal that the addition of nano-hematite in the geopolymer concrete provides better insight, for the benefit of academic and fundamental research, and promotes its practical application in the construction industry. Samen et al. [8] used non-calcined laterite containing hematite and goethite in order to investigate the impact of iron ions contained in laterite on the engineering properties of the Portland cement paste. The examination of the X-ray patterns of the final products indicate the presence of goethite. On the other hand, Fekoua et al. [9] used non-calcined goethite-rich aluminosilicate for the preparation of geopolymer materials. They reported that goethite reacts with sodium waterglass during geopolymerization, leading to the formation of fayalite and hinsingerite. Davidovits and Davidovits [10] reported that the impact of iron oxyhydroxides such as goethite on the properties of geopolymer materials seems dubious and that oxides such as hematite and magnetite would seem more favourable. The reaction of goethite with silicate described by Fekoua et al. [9] could be ascribed to the addition of rice husk ash and the fine particles of quartz in the system. Hence, the investigation of the best iron mineral for the increment of the compressive strength of geopolymer materials could be attractive.

This investigation aims to find, with certain additions of the iron minerals hematite, magnetite and goethite, possible systematic trends for upgrading or downgrading the compressive strength of geopolymer materials. Analytical techniques such as X-ray diffractometry, Fourier Transform infrared spectroscopy, scanning electron microscope and the compressive strength values measurement were used to monitor some changes in the geopolymer materials as well as the amorphous and crystalline phase contents. 

## 2. Results and Discussion

### 2.1. Characterisation of Starting Materials

#### 2.1.1. X-ray Patterns 

**Figure 1:**
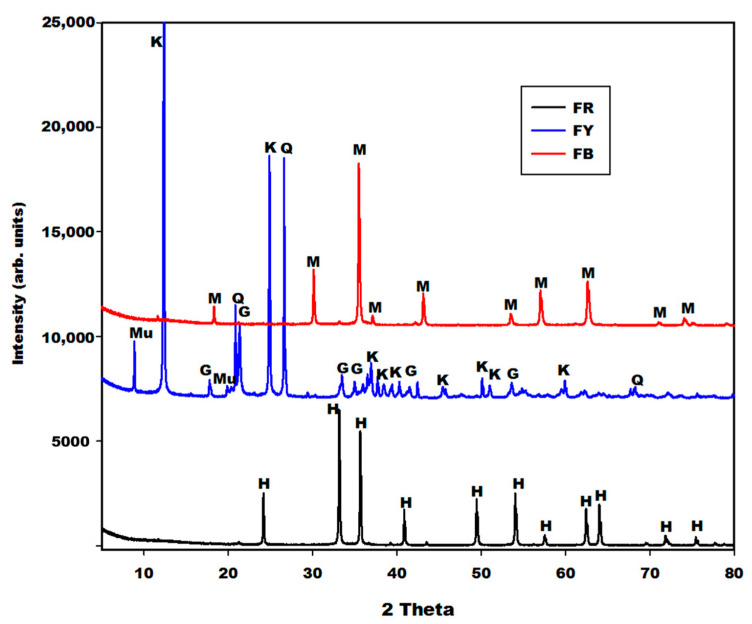
X-ray patterns of different oxides (FR, FB and FY). Q, H, M, G, Mu and K denotes the peaks of quartz, hematite, magnetite, goethite, muscovite and kaolinite, respectively.

**Figure 2:**
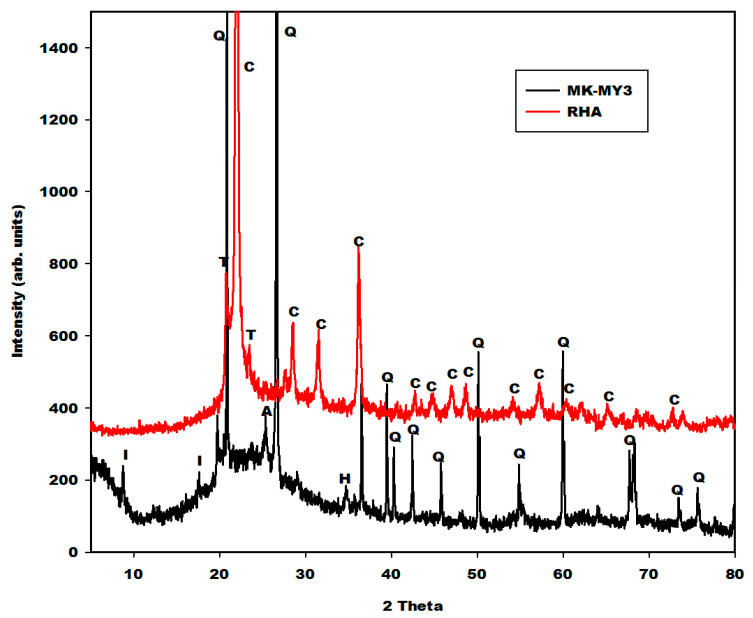
X-ray patterns of rice husk ash (RHA) and metakaolin (MK-MY3). I, Q, A, H and C, T referred to the peaks of illite, quartz, anatase, hematite, cristobalite and tridymite, respectively.

The X-ray patterns of different iron minerals (FR, FB and FY) are depicted in Figure 1, whereas those of rice husk ash (RHA) and metakaolin (MK-MY3) are shown in Figure 2. The X-ray patterns of different iron minerals such as FR (red powder) and FB depict the peaks of hematite (α-Fe_2_O_3_) and magnetite (Fe_3_O_4_), respectively. The sample FY (yellow powder) consists of goethite or oxyhydroxide (α-FeO(OH)) (26 wt.%), kaolinite (35 wt.%), quartz (33 wt.%) and muscovite (5 wt.%), according to Rietveld phase analysis. This shows that FR, FB and FY are associated with hematite, magnetite and goethite, respectively. Using the TOPAS package, the broadening of the peak profiles reveal average crystal sizes of 120 nm, 80 nm and 40 nm for hematite, magnetite and goethite, respectively (Lorentzian peaks, strain not considered). The oxidation state of iron in the structure of hematite and goethite is iron(III), whereas the one in the structure of magnetite is the combination of iron(III) and iron(II). The X-ray pattern of rice husk (RHA) indicates the peaks of cristobalite together with some minor tridymite. Metakaolin (MK-MY3) shows the peaks of illite, quartz, anatase and traces of hematite (Figure 2). Besides these minerals, both diffractograms show the broad hump structure from 18 to 38° (2θ) for RHA and 15 to 35° (2θ) for metakaolin. This halo diffraction is attributed to the amorphous silica and amorphous aluminosilicate phases in the structure of rice husk ash and metakaolin, respectively. 

#### 2.1.2. Infrared Spectra

**Figure 3:**
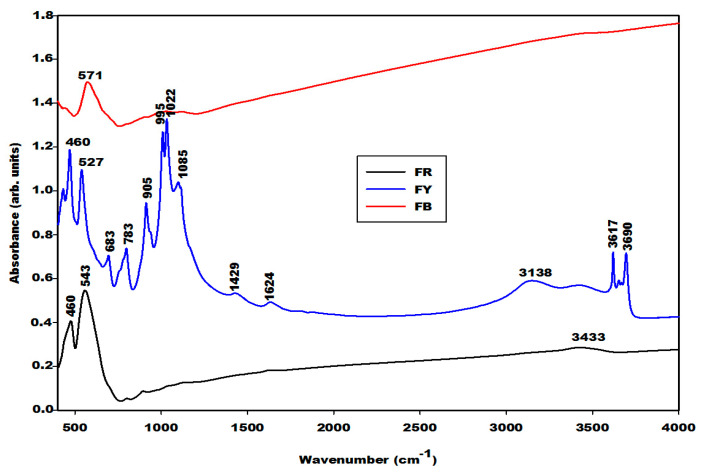
Infrared spectra of different iron oxide samples (FR, FB and FY).

The infrared spectra of the iron mineral samples (FR, FB and FY) are shown in Figure 3. The infrared spectrum of FR presents three absorption bands at 460, 543 and 3433 cm^−1^. According to Schwertmann and Cornell [11], Andrate et al. [12] and Deligöz et al. [13], the bands observed at 460 and 543 cm^−1^ are ascribed to the vibration modes of the Fe–O characteristics of hematite. The one of FB also shows three bands at 420, 571 cm^−1^, and the one with the lowest intensity is at about 3433 cm^−1^. The vibration modes of Fe–O were observed at 420 and 571 cm^−1^, which are characteristic of magnetite [11,12]. The absorption band at 3433 cm^−1^ on the infrared spectra of FR, FB and FY is assigned to the OH group stretching modes [14]. The absorption band at 3138 cm^−1^ presents only on the spectrum of FY is attributed to the Fe-OH stretching modes characteristic of goethite, and the one at 1624 cm^−1^ is assigned to the bending vibration modes of water molecules [15]. The latter band that appears at 1643 cm^−1^ on the spectra of metakaolin and rice husk ash indicates the presence adsorbed water. The absorption bands at 3617 and 3690 cm^−1^ on the spectrum of FY have been ascribed to the inner and external hydroxyl groups of kaolinite [16]. A rather close agreement is observed between the spectrum of sample FY and that of kaolinite. Therefore, the contribution of goethite is hard to see here in the IR-spectra. 

**Figure 4:**
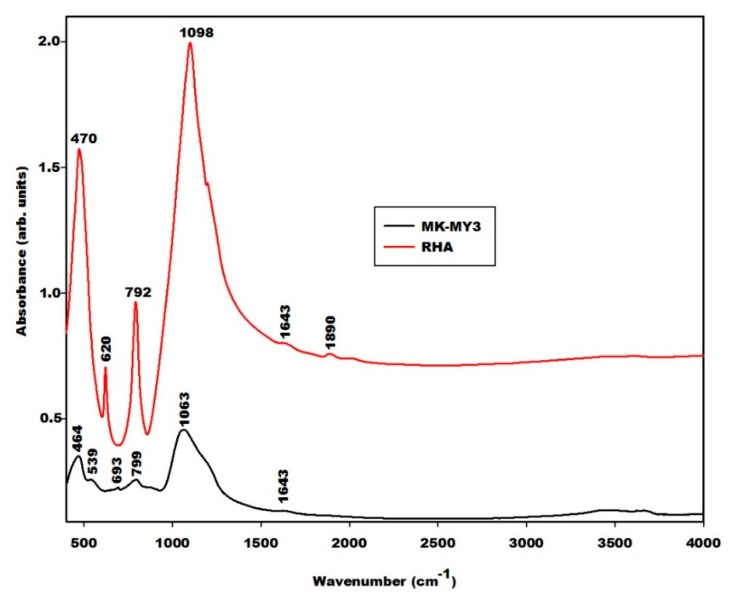
Infrared spectra of rice husk ash (RHA) and metakaolin (MK-MY3).

Figure 4 shows the spectra of rice husk ash (RHA) and metakaolin (MK-MY3). Those peaks that appear at 464 and 470 cm^−1^ on the infrared spectra of metakaolin and rice husk ash, respectively, are associated with the vibration modes of Si–O. The one at 539 cm^−1^ on the infrared spectrum of metakaolin is related to the vibration modes of Si–O–Al and Fe–O bonds. The interpretation of the Fe–O peak here is supported by the X-ray pattern, which shows a trace of hematite in the metakaolin sample. The broad absorption peak with a maximum at 1063 cm^−1^ is the typical density of state effect of asymmetric Si–O vibrations of calcined kaolinite. The weaker peaks at 693 and 799 cm^−1^ also appear typically in calcined kaolinite. The spectrum of rice husk ash typically composes features of SiO_2_-glass and cristobalite [17,18]. The main peak at 1098 cm^−1^ is ascribed to the asymmetric vibration modes of Si–O bonds. 

### 2.2. Characterisation of Geopolymer Materials

#### 2.2.1. X-ray Diffractograms, Crystalline and Amorphous Phases Content

**Figure 5:**
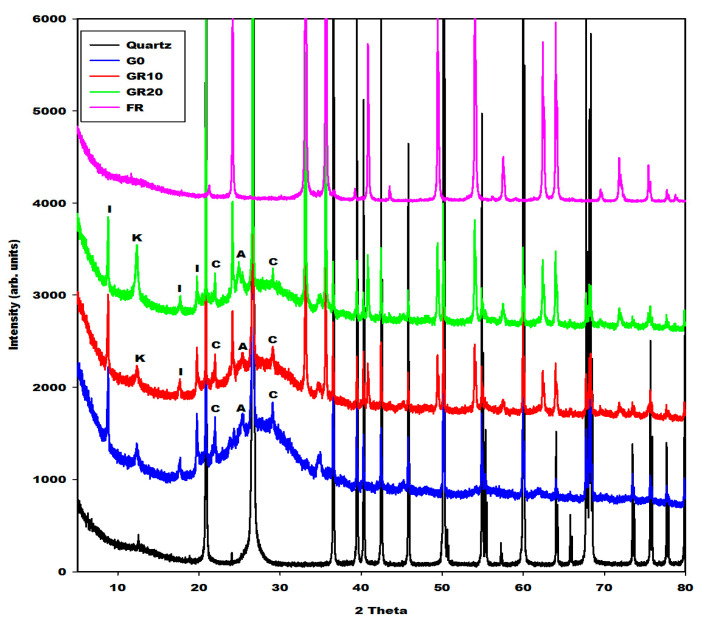
X-ray patterns of geopolymer materials (G0, GR10, GR20, quartz and FR: Hematite). I, K, A and C denotes the peaks of illite, kaolinite, anatase and cristobalite, respectively.

**Figure 6:**
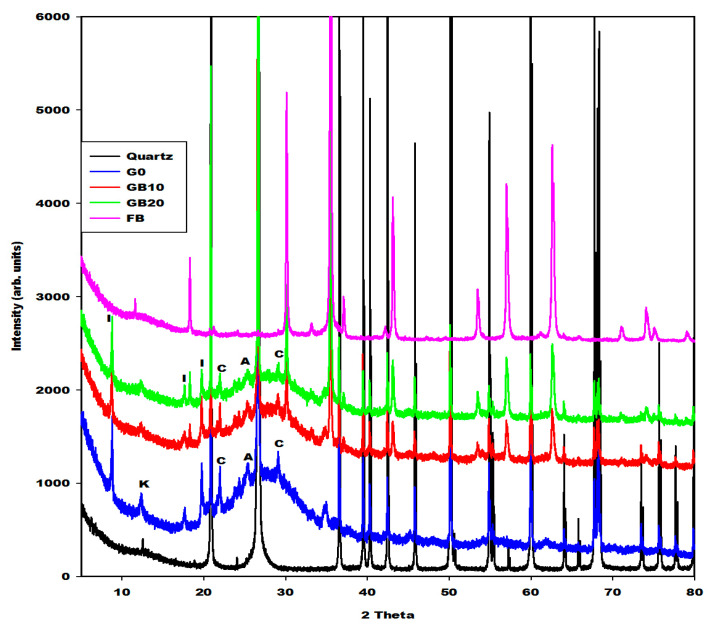
X-ray patterns of geopolymer materials (G0, GB10, GB20, quartz and FB: Magnetite). I, K, A and C denotes the peaks of illite, kaolinite, anatase and cristobalite, respectively.

**Figure 7:**
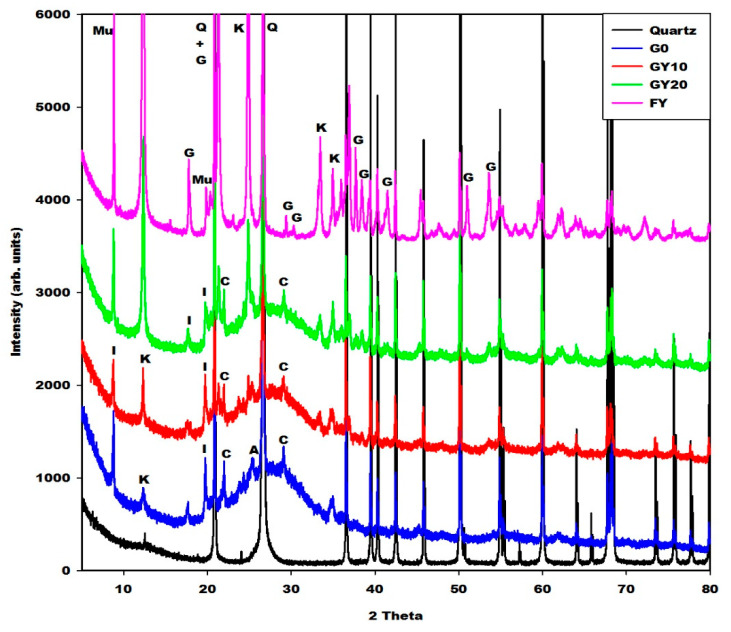
X-ray patterns of geopolymer materials (G0, GY10, GY20, quartz and FY). Q, I, K, A and C denotes the peaks of quartz, illite, kaolinite, anatase and cristobalite, respectively.

Figure 5, Figure 6 and Figure 7 depict the X-ray patterns of geopolymer materials from metakaolin-FR (GR10 and GR20), metakaolin-FB (GB10 and GB20) and metakaolin-FY (GY10 and GY20), respectively. The control geopolymer material has been denoted G0, and metakaolin has been substituted by 10 and 20 wt.% of different iron mineral samples. The peaks of illite, quartz, anatase, cristobalite and the residual kaolinite are observed on the X-ray patterns of all geopolymer materials. In addition to these crystalline minerals, the X-ray patterns show the broad hump structure between 18 and 40° (2θ), attributed to the amorphous aluminosilicate phase or gels in the network of geopolymer materials. It is worth mentioning that this amorphous phase appears between 15 and 38° (2θ) on the X-ray pattern of metakaolin (Figure 2). The shift of this broad peak is characteristic of the depolymerization process of metakaolin or iron-metakaolin followed by the polycondensation reaction, entailing the formation of the geopolymer binders. Besides these phases, the X-ray patterns of geopolymer materials from metakaolin-FR and metakaolin-FB (Figure 5 and Figure 6) indicate rather clearly the peaks of hematite and magnetite, respectively. For the geopolymer samples GY10 and GY20 (Figure 7), the goethite related peaks are observed, albeit with relatively lower intensities. Rietfeld refinements reveal in all cases the same average crystal sizes as obtained for the raw iron minerals.

The crystalline and amorphous phase (gels) contents in the geopolymer materials G0, GR10, GR20, GB10, GB20, GY10 and GY20, as obtained in the first step by integration, are depicted in Table 1. These data show that the X-ray amorphous contribution in G0, GR10, GR20, GB10, GB20, GY10 and GY20 are 49.4, 38.6, 35.4, 43.5, 39.9, 45.1 and 42.0%, respectively. It can be observed that the X-ray amorphous contribution in the geopolymer materials systematically decreases when the iron minerals increase from 0 to 20 wt.%. This is obvious because the iron minerals used for the substitution of metakaolin remain crystalline minerals (Figure 1). As the liquid-to-solid ratio remains almost constant, this does not lead to higher contents of gel. The reduction in the amorphous phase content when the iron minerals content increases in the system is confirmed by the reduction in the intensity of the broad hump network that appears from 18 to 40° (2θ) on the X-ray patterns of geopolymer materials (Figure 5, Figure 6 and Figure 7). The decrease in the amorphous-phase content seemed to be more pronounced when metakaolin was substituted by 10 and 20 wt.% of hematite (FR). This could be related to the higher degree of crystallinity of hematite or to the formation of the crystalline network in the structure of GR10 and GR20, which is not detected on their X-ray patterns compared to those of GB10, GB20, GY10 and GY20. On the other hand, the integration of the X-ray scattered intensities ignores any element-specific contributions. Therefore, using structural and element specific contributions, as included in the Rietveld method of phase quantification using an internal standard, could reveal more realistic quantifications, which were not further considered here. 

#### 2.2.2. Infrared Spectra

**Figure 8:**
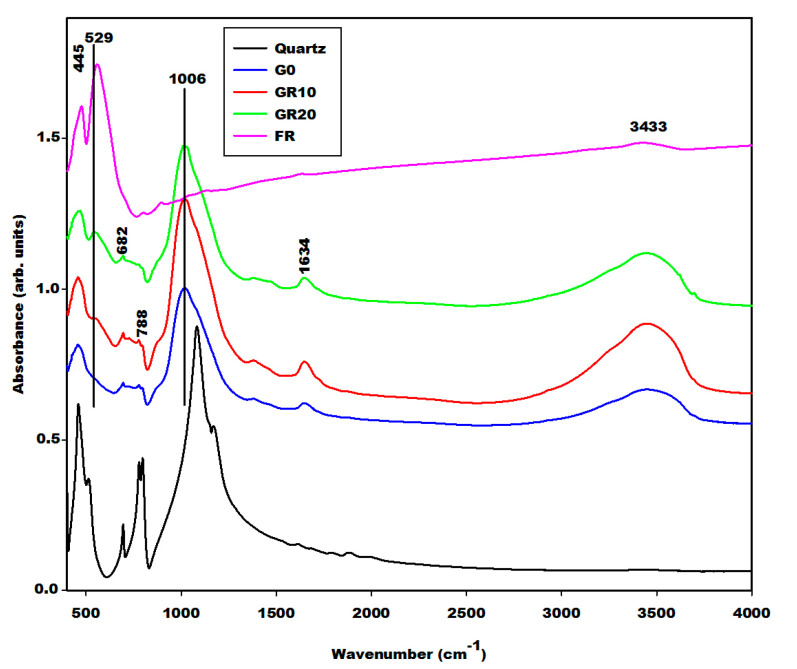
Infrared spectra of geopolymer materials (G0, GR10, GR20, quartz and FR: Hematite).

**Figure 9:**
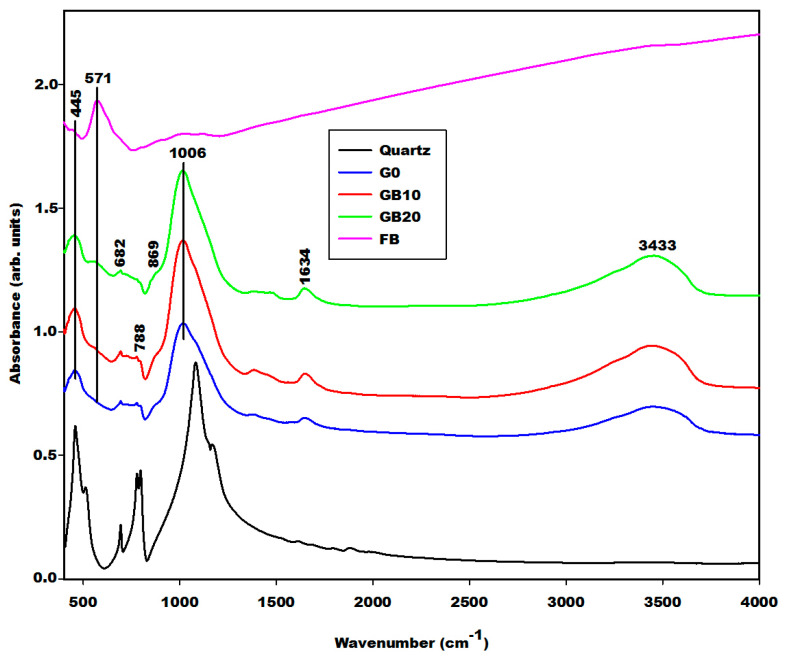
Infrared spectra of geopolymer materials (G0, GB10, GB20, quartz and FB: Magnetite).

**Figure 10:**
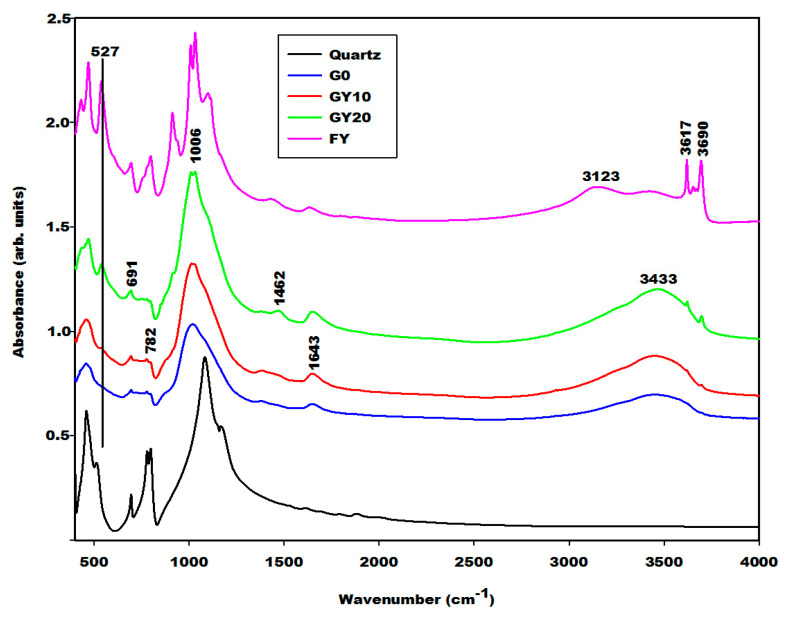
Infrared spectra of geopolymer materials (G0, GY10, GY20, quartz and FY: Goethite and kaolinite).

The geopolymer material both with and without iron minerals show some changes detected by the Fourier Transform Infrared spectroscopy in Figure 8, Figure 9 and Figure 10. The infrared spectra of geopolymer materials after iron mineral addition (10 and 20 wt.% of FR, FB and FY) show absorption bands at 529 cm^−1^, 571 cm^−1^ and 527 cm^−1^, as denoted in Figure 8, Figure 9 and Figure 10, respectively. Peaks between 571 and 529 cm^−1^ can be attributed to vibration modes of Fe–O–Si, as discussed in the literature [19,20,21,22,23,24,25]. According to Essaidi et al. [4], the iron mineral modifies the formation of the geopolymer material, leading to the formation of different specific networks with the binder. It is also clearly observed that the infrared spectrum of the geopolymer material without the addition of iron mineral does just show an absorption wing between 520 cm^−1^ up to 600 cm^−1^, but not a peak, as observed for the 10 and more intensive values for 20% of the samples. This could indicate that iron minerals added to the metakaolin could react with sodium waterglass during the geopolymerization process. However, we may also give a different explanation. A reaction of Fe^3+^ from hematite for the formation of the poly(ferro-silico-aluminate) network cannot be easily supported by the present results. It is observed that the samples GR10 and GR20 show an additional peak at 529 cm^−1^ (Figure 8) compared to G0. This peak can be related to the presence of hematite as having shifted from 543 cm^−1^ due to superimposition. We checked this by adding up the appropriate spectra of hematite and G0, reproducing the peak position as observed experimentally. This showed us that the observed peak shift cannot be taken as an indication of incorporation of Fe–O units in the geopolymer network. The absorption bands of magnetite and the FY sample at 571 and 527 cm^−1^ (Figure 3) remain unchanged on the spectra of GB10 and GB20 (Figure 9) and those of GY10 and GY20 (Figure 10), respectively. This suggests that magnetite and hematite do not participate in the geopolymerization process. For GY10, and more clearly for GY20, there are additional peaks that can be related to the unreacted kaolinite, but there is no clear indication of the effect of goethite observed as for the FY sample (Figure 3).

All geopolymer spectra show silanol groups (Si–OH) identified at around 869 cm^−1^. A significant broad band centred at the wavenumber of around 1006 cm^−1^ on the infrared spectra of geopolymer materials, which corresponds to the density of states of the Si–O asymmetric stretching of the various [SiO_4_]-units of the geopolymer network. This band appears at the wavenumber of 1063 cm^−1^ on the spectrum of metakaolin (Figure 4). It was shifted to a lower wavenumber on the spectra of geopolymer materials owing to the depolymerization of metakaolin in the presence of sodium waterglass. Due to the consumption of NaOH from the waterglass, poly-siloxo chains are formed, which become crosslinked via sialate and siloxo units in the following. A significant incorporation of Fe in the network may also be ruled out here due to rather precisely the same position and shape in the density of Si–O states for the G0-sample and the G10 and G20 samples.

The absorption bands with lower intensity at 3617 and 3690 cm^−1^ on the infrared spectrum of geopolymer material after 20 wt.%, GY20 and somewhat weaker for GY10 (Figure 10) are related to kaolinite in the FY sample, which remains unreacted during geopolymerization. The quartz is identified at 682–691 cm^−1^ and 782–788 cm^−1^ on the infrared spectra of geopolymer materials. These bands were also attributed to the vibration modes of Si–O–Fe and Fe–O–Al, respectively [19,20,21,22,23,24,25,26,27,28], which cannot be supported by the present results. 

#### 2.2.3. Micrography Image Investigations

**Figure 11:**
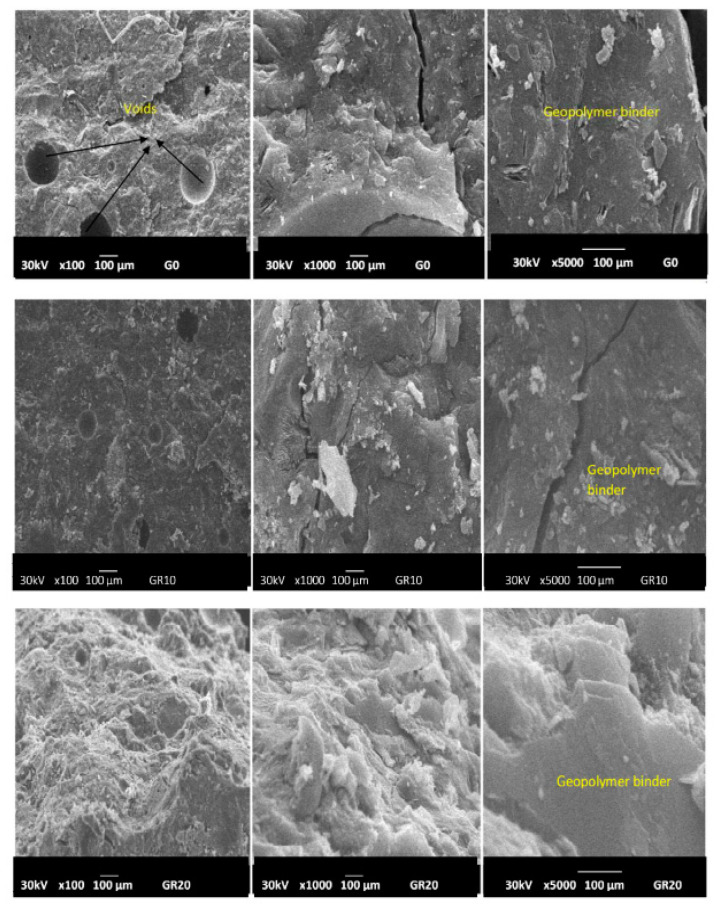
Micrograph images of geopolymer materials without (G0) and with the addition of hematite (GR10, GR20) with magnifications of x100, x1000, x 5000 (from **left** to **right**).

**Figure 12:**
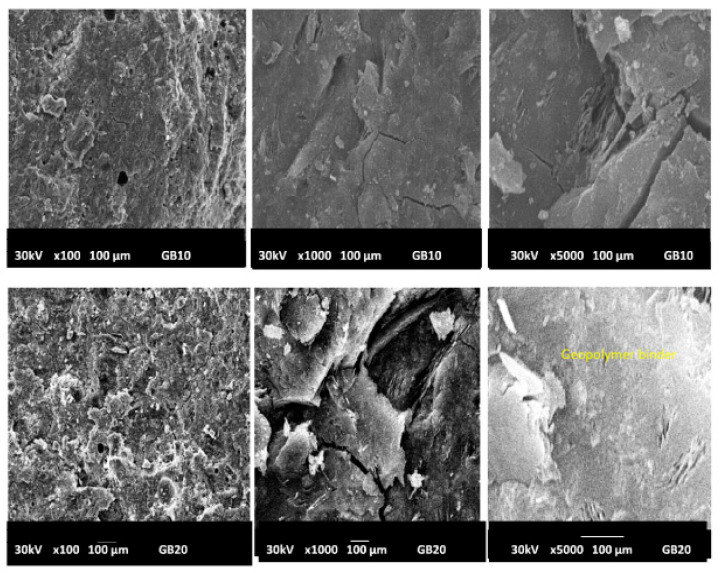
Micrograph images of geopolymer materials with the addition of magnetite (GB10, GB20) with magnifications x100, x1000, x5000 (from **left** to **right**).

**Figure 13:**
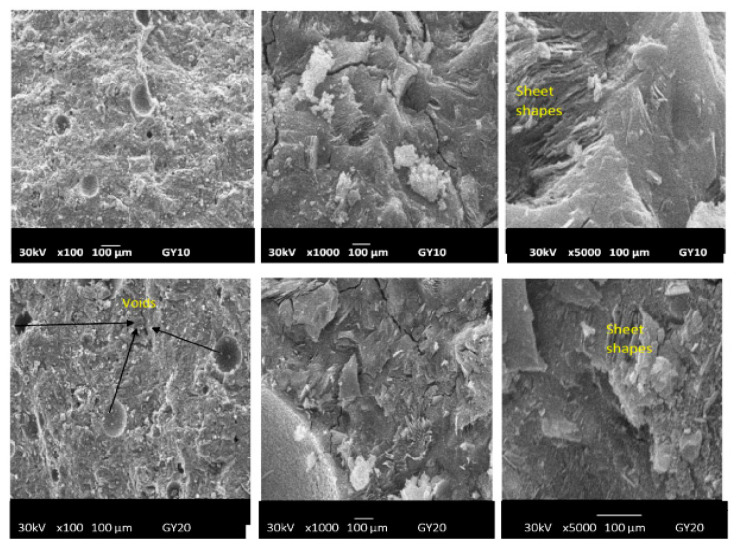
Micrograph images of geopolymer materials with addition of goethite containing kaolinite. (GY10, GY20) with magnifications of x100, x1000, x5000 (from **left** to **right**).

The morphologies of geopolymer materials G0, GR10, GR20, GB10, GB20, GY10 and GY20 examined at magnifications of 100×, 1000× and 5000× are shown in Figure 11, Figure 12 and Figure 13. The micrographs of the prepared geopolymer materials show a relatively compact consistency, indicating that the crystalline phases present in the geopolymer materials are embedded in their networks. This could affect the compressive strength of the geopolymer materials, as discussed below. Some samples observed at a magnification of 100× present some voids and air bubbles, which could lead to substantially smaller compressive strengths of the geopolymer materials. The cracks appear on the micrographs observed at a magnification of 1000× and 5000×. These cracks generally appeared at the time the compressive strengths were measured. Although most regions of the geopolymer materials GY10 and GY20 observed at a magnification of 5000× are a denser matrix, their micrograph images show some sheet shapes associated with the kaolinite also observed in the X-ray patterns of these geopolymer materials (Figure 7). The presence of the kaolinite in the samples of GY10 and GY20 could limit the binding system and therefore decrease their compressive strength values, as discussed below. 

**Figure 14:**
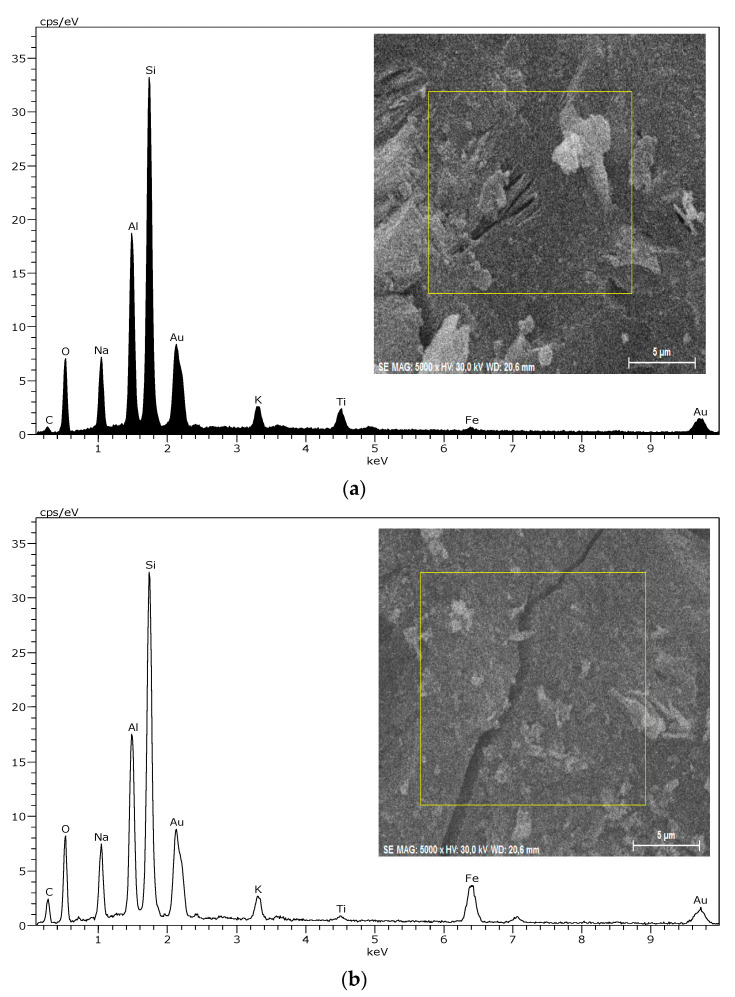

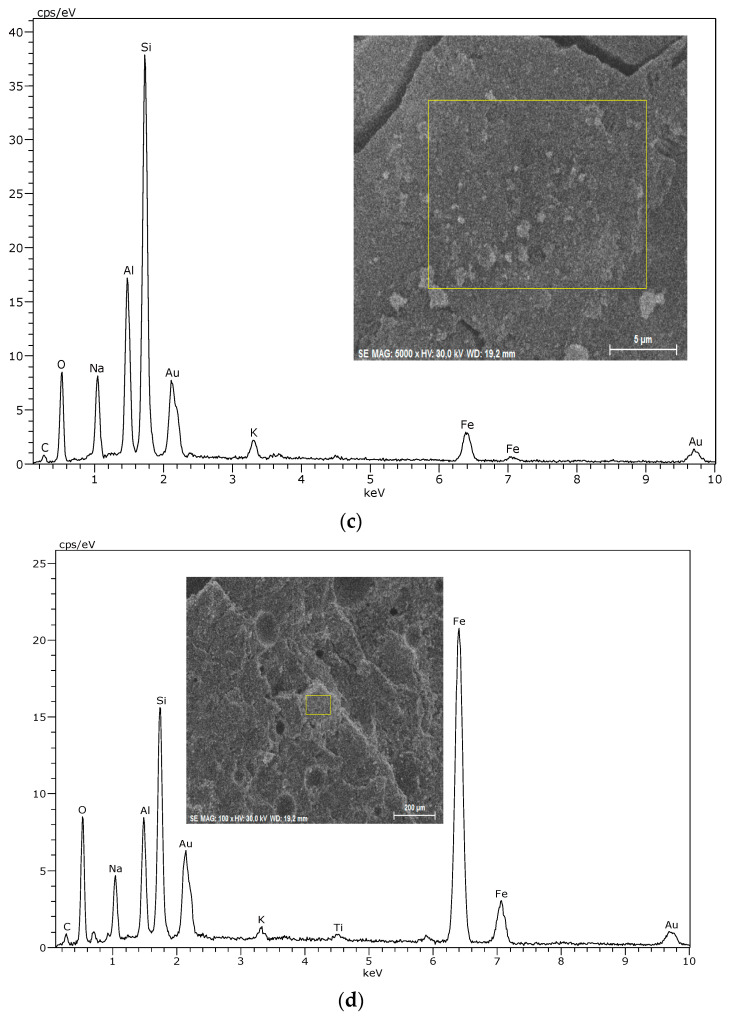
EDS spectra of areas of samples G0 (**a**), GR10 (**b**) and GR20 (**c**) as indicated in the inserted micrographs by the red rectangles (taken with magnification 5000×). With an average of quantification of 5, such areas of each sample are given in Table 3. (**d**) EDS spectrum of GR20 taken at a magnification of 100× and focused on a smaller area with a higher iron content, suggesting a localization of hematite.

Typical results of the EDS analysis of the geopolymer materials G0, GR10 and GR20 are given in Figure 14. The spectra of G0 indicate that this geopolymer material is mainly composed of Si, Al and Na, which is ascribed to the geopolymer gel. For the geopolymer materials GR10, GR20, the addition of hematite shows increased Fe peak intensities. However, it is also possible to find areas without Fe, with increased Si due to quartz content and increased Fe due to the presence of hematite. For example, it is shown in Figure 14d for the sample GR20 with higher magnification and focused on an area of higher Fe content. Similar results were obtained also for samples GB10, GB20 and GY10 and GY20. This means that the iron minerals are not solved to a large extent by the waterglass. It shows that the geopolymer materials from the addition of iron minerals are heterogeneous materials. However, a systematic EDX analysis using the same areas at a magnification of 5000× reveal rather reliable Fe contents, which show similarly increased values for GR10 to GR20, GB10 to GB20 and GY10 to GY20 (Table 2). For GY10 and GY20, the Fe contents are smaller due to the lower content of Goethite in the raw material added.

#### 2.2.4. Compressive Strengths

**Figure 15:**
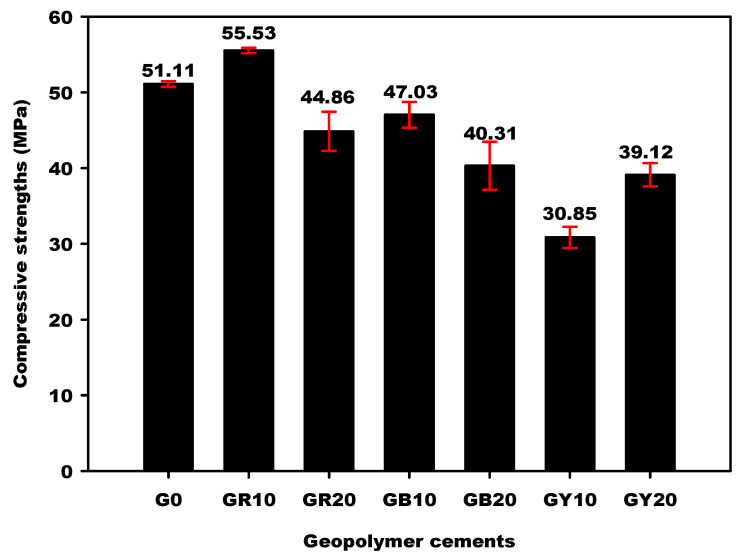
Compressive strengths of geopolymer materials.

The influence of different iron minerals on the compressive strengths of the metakaolin-based geopolymer materials is visualized in Figure 15. This figure shows that compressive strengths of the geopolymer materials G0, GR10, GR20, GB10, GB20, GY10 and GY20 are 51.11, 55.53, 44.86, 47.03, 40.31, 30.85 and 39.12 MPa (error bars as indicated), respectively.

It can be observed that the compressive strengths of geopolymer materials from the substitution of metakaolin by 0, 10 and 20 wt.% of FR (hematite) increase moderately from 51.11 to 55.53 MPa, followed by a decrease from 55.53 to 44.86 MPa. Those containing 0, 10 and 20 wt.% of FB (magnetite) decreased from 51.11 to 40.31 MPa with increasing iron mineral content. On the other hand, the compressive strengths of the geopolymer materials containing 10 wt.% of goethite decreased from 55.11 to 30.85 MPa and increased from 30.85 to 39.12 MPa when 20 wt.% of goethite was added to the system. The lower compressive-strength values of geopolymer materials containing goethite could be ascribed to the presence of unreacted kaolinite in their structure, which inhibits the geopolymerization process. The presence of kaolinite is supported by the XRD and SEM results of GY10 and GY20 (Figure 7 and Figure 13). It can be seen that the compressive strengths of geopolymer materials containing hematite and magnetite are higher compared to the ones containing goethite. SEM results showed, for hematite- and magnetite-containing geopolymers, a more compact and denser matrix compared to the samples with goethite added, which supports the better mechanical properties. This finding also agrees to some extent with Davidovits and Davidovits [10], who reported that iron oxides such as hematite and magnetite are more favourable for geopolymer materials synthesis than that of goethite. However, in our case, the compressive strengths of geopolymer materials containing magnetite decrease as well. Only when metakaolin was replaced by 10 wt.% of hematite was the binder reduced in the system, but the compressive strengths increase. The increase in the compressive strengths could be related to the fact that the particles of hematite do not react during the geopolymerization reaction but may promote a better geopolymerization process into a compact, integrated morphology. This could hinder the crack propagation, similarly to a toughening mechanism proposed by Ghazanlou et al. [3] for the gel of cement-micro/nano Fe_3_O_4_ composites. Our results are contrary to the work of Choi and Lee [29] and Essaidi et al. [4], who reported that the addition of 5 wt.% of hematite inhibits better mechanical properties owing to the interaction between the specific network and the binder. On the other hand, the work here confirms the results obtained by Davidovits and Davidovits [10], insofar as metakaolin containing hematite to some extent causes higher compressive strengths compared to metakaolin-based geopolymers without any hematite. According to Zailani et al. [5], an iron silicate binder could be formed by a chemical reaction between silicate and iron(III) in addition to the geopolymer networks. Meanwhile, Kaze et al. [23] and Davidovits and Davidovits [10] identified the ferro-sialate in the structure of geopolymer materials and reported that this compound leads to an increase in the compressive strength and also contributes to the densification of geopolymer networks. To our understanding, the presence of hematite to a limited extent improves the gel-formation and interaction between crystal additions as a glue and therefore increases the compressive strength of the geopolymer materials. This assertion has been supported by the amorphous and crystalline phases content reported in Table 1. It appears in this table that GR10 has only 38.6 wt.% of amorphous phase content and 61.4 wt.% of crystalline phase content, whereas GB10 and GY10 have 43.5 and 45.1 wt.% of amorphous phase content, respectively. This could indicate that the particles of hematite that do not participate in the geopolymerization process act as a filler and reinforce the microstructure of the geopolymer materials despite their low amorphous-phase content. The higher compressive strength value of GR10 corroborates the findings of Hawa et al. [30], who reported that the higher crystallinity content entails a higher compressive strength. Alvarez-Ayuso et al. [31] and Al Kakni et al. [32] concluded that fly-ash-based geopolymers with increased crystallinity exhibited increased compressive strength. The reduction in the strength after the incorporation of 20 wt.% of hematite could be ascribed to the excessive hematite in the system, which prevents the geopolymerization reaction. The compressive strength values of geopolymer materials containing 10 and 20 wt.% of magnetite (GB10 and GB20) and FY-sample (GY10 and GY20) decrease despite their higher amorphous phase content (Table 1). This could be related to the fact that the incorporation of the magnetite or FY-composition in the geopolymer structures hinders the geopolymerization process. Nevertheless, the compressive strength values of the geopolymer materials containing FB and FY remain acceptable (Figure 15). Hematite (α-Fe_2_O_3_) and goethite (α-FeO (OH)) contain Fe^3+^, whereas magnetite (Fe_3_O_4_) contains Fe^2+^ and Fe^3+^. Dan et al. [33] and Lemougna et al. [34] concluded that the Fe^3+^ react with the geopolymer binder. A fine-dosed substitution of Al^3+^ by Fe^3+^ during the polycondensation reaction may increase the number of nucleation sites and therefore increases the compressive strength of the geopolymer materials from 51.11 to 55.53 MPa when 10 wt.% of hematite is added to the geopolymer materials. The decrease in the compressive strength of geopolymer materials when magnetite was used as an additive could be ascribed to the fact that Fe^2+^ of magnetite did not participate in the geopolymerization process [34] and Fe^3+^ ions in magnetite were affected by Fe^2+^ and cannot participate in the reaction [28]. The decrease in the compressive strengths from 51.11 to 30.85 MPa of the geopolymer materials when the sample FY is added to 10 wt.% could well be related to the combined effect of adding uncalcined kaolinite, goethite, quartz and muscovite. The kaolinite cannot be activated by the solution and may weaken the composite. Otherwise, the increase in the compressive strength from 30.85 to 39.12 MPa with 10 wt% to 20 wt.% of goethite is in agreement with the findings of Liao et al. [35], Heikal and Ibrahim, [36] and Saleghi-Nik et al. [37], who reported that the inclusion of the nano clay in the structure of cementitious materials positively influences the mechanical properties.

## 3. Conclusions

Three iron minerals were used as additives to investigate their influences on the compressive strengths, the amorphous or crystalline phases content and the microstructural properties of metakaolin-based geopolymer materials. The X-ray patterns and the infrared spectra of red, black and yellow minerals showed that they are hematite (α-Fe_2_O_3_), magnetite (Fe_3_O_4_ or FeO. Fe_2_O_3_) and goethite (α-FeO(OH)), respectively. Metakaolin was replaced by 0, 10 and 20 wt.% of each additive, and the sodium waterglass from rice husk ash was used as a hardener. The compressive strength of the geopolymer material without additive was around 51 MPa. There was an increase from 51 to 55 MPa and a decrease from 55 to 45 MPa when metakaolin was substituted by 10 and 20 wt% of hematite, respectively. The crystalline phase content is higher in the structure of geopolymer materials using hematite as an additive compared to the ones using the magnetite and FY sample. The X-ray patterns of geopolymer materials indicate that the intensity of the broad hump structure that appears between 18 and 40° (2θ) decreases with increasing iron mineral content. The micrograph images of geopolymer materials show that the crystalline phases are embedded in the matrix. It is concluded that amongst the iron minerals used in this work, hematite of sizes in the range of 120 nm with contents of about 10 wt% could be used to improve the compressive strength of the geopolymer materials. Intensified investigations including the crystal size dependencies of the mineral additions similar to that carried out for cement-micro/nano Fe_3_O_4_ composites by [3] could be worthwhile. It is without any doubt that calcined iron oxide containing clays and as-prepared sodium silicate solutions using rice ask ash are suitable to displace the use of ordinary Portland cement for any usual building construction.

## 4. Materials and Experimental Methods

### 4.1. Materials

The kaolin used in this work was denoted MY3. It was collected from the locality of Mayouom in the West region of Cameroon. This kaolin has been studied and described by Njoya et al. [38]. They reported that this aluminosilicate source is mainly constituted of kaolinite associated with a minor content of illite, quartz, anatase and hematite. The raw kaolin, once harvested, was broken into small sizes using a hammer. The obtained material was dried in air for 24 h and pulverized in the ball mill (MGS, Srl) for 30 min. The powder of kaolin was calcined at 700 °C using a heating and cooling rate of 5 °C/min in the programmable electric furnace for 4 h. The obtained metakaolin has already been described by Tchakouté et al. [39,40] and is denoted as MK-MY3. Three iron minerals with different colours such as red, yellow and black, called FR, FY and FB, respectively, were purchased by the Serraciments company located in Barcelona, Spain. The company denoted UNVDA (Upper Nyoung Valley Development Association) situated in Ndop in the North-West region of Cameroon provided white rice husk ash, which is already calcined in an open air. This white silica source was ground in the same condition as kaolin and the powder obtained was denoted RHA. The chemical compositions of the kaolin and rice husk ash are presented in Table 3. Rice husk ash has already been used and is described by Melele et al. [41], Mabah et al. [42] and Tchakouté et al. [40]. NaOH pellet was provided by the laboratory-grade granules (96 wt.%, Sigma Aldrich, Milano, Italy).

**Table 3:** Chemical compositions of kaolin (MY3) and rice husk ash (RHA), wt.%.

	Samples	MY3 Njoya et al. (2006)	RHA Melele et al. (2019), Mabah et al. (2019)
Oxides			
MgO		/	0.28
Al_2_O_3_		33.29	0.58
SiO_2_		46.61	93.20
K_2_O		0.94	3.05
CaO		/	0.57
TiO_2_		3.96	0.03
SO_2_		0.05	/
Fe_2_O_3_		1.46	2.20
P_2_O_5_		0.40	/
Others		/	1.78
LOI		13.97	1.2

### 4.2. Experimental Methods

#### 4.2.1. Preparation of Sodium Waterglass from Rice Husk Ash and Geopolymer Materials

Sodium waterglass containing the molar ratio SiO_2_/Na_2_O equal to 1.6 has been prepared using rice husk ash as the silica source and sodium hydroxide solution as the Na_2_O source. Sodium hydroxide solution with a molar concentration of 5 M was obtained by dissolving a sodium hydroxide pellet in distilled water. The prepared sodium hydroxide solution was mixed with rice husk ash (100 g), and the mixture was heated for about 1 h at 100 °C using a magnetic stirrer to obtain a solution called sodium waterglass. 

For the preparation of geopolymer materials, metakaolin was firstly substituted by 0, 10 and 20 wt.% of each iron oxide in order to have iron-metakaolin. Each powder obtained was mixed with the prepared sodium waterglass. The mass ratio of the liquid/solid of the geopolymer material without iron mineral addition and those containing iron minerals were 0.97 and 1.02, respectively. The obtained mixture was blended for about 5 min, and the obtained pastes of each composition were moulded in the cube moulds with dimensions of 40 mm × 40 mm × 40 mm. The moulded samples were cured for 24 h at the ambient temperature of the laboratory before demoulding. After demoulding, the compositions were sealed in the plastics and left in the laboratory for 28 days before their compressive strength measurements. The control geopolymer materials (without iron mineral addition) are denoted G0. The geopolymer materials from the incorporation of 10 and 20 wt.% of red iron mineral are named GR10 and GR20, respectively. Those from the inclusion of 10 and 20 wt.% of black iron mineral are designated as GB10 and GB20, respectively, whereas the ones of yellow iron mineral are called GY10 and GY20, respectively.

#### 4.2.2. Methods of Characterization of Raw Materials and Metakaolin-Based Geopolymer Materials

The influence of the red, yellow and black iron minerals on the properties of geopolymer gels was monitored by measuring their compressive strengths and investigating their microstructural properties (infrared spectroscopy analysis, the powder X-ray diffractometry and the scanning electron microscope).

The compressive strength values of the geopolymer materials were determined after 28 days on the samples sealed in the plastics and maintained at room temperature in the laboratory according to the DIN 1164 standard. A loading rate was kept constant at 0.500 MPa/s. They were measured using an automatic hydraulic press with a 250 kN capacity (Impact Test Equipment Limited, UK KA20 3LR, Stevenston, United Kingdom). After the compressive strengths were tested, the fragments of all samples were collected, and one part was finely crushed in the porcelain mortar. The obtained powders were used to record the X-ray diffractograms and infrared spectra. Others fragments were used to observe the morphologies of the samples. 

The X-ray diffractograms were registered on the Bruker D8 (Karlruhe, Germany) Advance equipped with LynXeye XE T detector detecting CuKα1,2 in Bragg Brentano geometry. The 2θ range is from 5 to 80° for 1 h with 0.01 2θ steps. The crystalline phases present in the structure of different samples were identified using X’Pert HighScore Plus software (version 3.0.0.123). The crystalline and amorphous phase contents were estimated by correcting for the experimental background obtaining the total intensity, S(tot) and then integrating only the crystalline part, S(Cryst) and calculating S(amorphous) = S(tot) − S(cryst). For this purpose, Opus software was used. Phase contents of the sample FY were refined using the Rietveld method (Topas 6.0, Bruker). Average crystals sizes were obtained using refinements of the raw iron minerals and these phases obtained in the geopolymer.

The infrared spectra were measured by FTIR (Bruker Vertex 80 v, Bremen, Germany) using the KBr method. In this method, about 200 mg of KBr was mixed with around 1 mg of the sample, and the whole was blended in an agate mortar. The mixture was pressed at 100 kN using a hydraulic press (ENERPAC P392, San Francisco, CA, USA) in order to obtain the pellets. Each pellet was used to record a spectrum with the resolution of 2 cm^−1^ and 16 scans, and the data were collected using OPUS software. 

The other fragments of the geopolymer materials collected after the compressive strength measurement were prepared for morphological investigation. The preparation of the specimens consists of coating the samples with gold in the chamber. The specimens with gold coating were used to observe the micrograph images of each sample at different magnifications 100×, 1000× and 5000×. This was done using a JEOL JSM-6390A Scanning Electron Microscope (SEM), (Tokyo, Japan) with an acceleration voltage of 30.0 kV.

## Figures and Tables

**Table 1:** Amorphous and crystalline phase contents (wt.%).

Specimens	Amorphous Phase Contents (wt.%)	Crystalline Phase Contents (wt.%)
G0	49.4	50.6
GR10	38.6	61.4
GR20	35.4	64.6
GB10	43.5	56.5
GB20	39.9	60.1
GY10	45.1	54.9
GY20	42.0	58.0

**Table 2:** Chemical composition from EDS analysis (Atom%, average of 5 fixed areas using magnification 5000×).

Specimens	O_2_	Na	Al	Si	Ti	K	Fe
G0	60.57	8.00	10.89	19.60	0.34	0.52	0.08
GR10	60.49	7.32	11.41	17.78	1.34	0.37	1.27
GR20	57.44	11.07	8.81	16.18	0.08	0.51	5.93
GB10	59.08	10.06	9.10	18.92	0.02	0.60	2.21
GB20	60.55	6.45	5.74	21.61	0.0	0.29	5.38
GY10	60.83	6.75	11.79	19.18	0.25	0.56	0.62
GY20	60.67	6.13	8.95	20.23	0.16	0.90	2.96

## Data Availability

Data obtained as described.
